# Aldehyde dehydrogenase 2*2 knock-in mice show increased reactive oxygen species production in response to cisplatin treatment

**DOI:** 10.1186/s12929-017-0338-8

**Published:** 2017-05-22

**Authors:** Jeewon Kim, Che-Hong Chen, Jieying Yang, Daria Mochly-Rosen

**Affiliations:** 1 Stanford Cancer Institute, Stanford University, School of Medicine, Stanford, CA 94305 USA; 2 Department of Chemical and Systems Biology, Stanford University, School of Medicine, Stanford, CA 94305 USA

**Keywords:** Aldehyde dehydrogenase, Cisplatin and Reactive Oxygen Species

## Abstract

**Background:**

The aldehyde dehydrogenase (ALDH) enzyme family metabolizes and detoxifies both exogenous and endogenous aldehydes. Since chemotherapeutic agents, such as cisplatin, generate cytotoxic aldehydes and oxidative stress, and chemoresistant cancer cells express high levels of ALDH enzymes, we hypothesized that different ALDH expression within cells may show different chemosensitivity. ALDH2 has the lowest *K*m for acetaldehyde among ALDH isozymes and detoxifies acetaldehydes in addition to other reactive aldehydes, such as 4-hydroxy-nonenal, malondialdehyde and acrolein produced from lipid peroxidation by reactive oxygen species (ROS). Thus, cells with an ALDH2 variant may sensitize them to these ROS-inducing chemotherapy drugs.

**Methods:**

Here, we used wild type C57BL/6 mice and ALDH2*2 knock-in mutant mice and compared the basal level of ROS in different tissues. Then, we treated the mice with cisplatin, isolated cells from organs and fractionated them into lysates containing mitochondrial and cytosolic fractions, treated with cisplatin again in vitro, and compared the level of ROS generated.

**Results:**

We show that overall ROS production increases with cisplatin treatment in cells with ALDH2 mutation. The treatment of cisplatin in the wild type mice did not change the level of ROS compared to PBS treated controls. In contrast, ALDH2*2 knock-in mutant mice showed a significantly increased level of ROS compared to wild type mice in tongue, lung, kidney and brain tissues without any treatment. ALDH2*2 mutant mice showed 20% of the ALDH2 activity in the kidney compared to wild type mice. Treatment of ALDH2*2 mutant mice with cisplatin showed increased ROS levels in the mitochondrial fraction of kidney. In the cytosolic fraction, treatment of mutant mice with cisplatin increased ROS levels in lung and brain compared to PBS treated controls. Furthermore, ALDH2*2 mutant mice treated with cisplatin showed increased cytotoxicity in the kidney cells compared to PBS treated mutant controls.

**Conclusions:**

These data indicate that deficiency in ALDH2 activity may contribute to increased cisplatin sensitivity and cytotoxicity by producing more ROS by the treatment. Based on these data, the amount of cisplatin used in patients may need to be adjusted based on their ALDH2 variant profile.

## Background

The current standard of care in cancer involves multiple modalities of treatment, including surgery, chemotherapy, and radiation. Cisplatin is currently one of the most commonly used chemotherapeutic agents for solid tumors [1]. However, cisplatin resistance and a significant incidence of toxic side effects (e.g., ototoxicity and nephrotoxicity) pose serious issues in using cisplatin [2, 3].

Cisplatin induces cytotoxicity partly by producing reactive oxygen species (ROS) and damaging DNA, which induces apoptosis, inhibiting further cell proliferation. The cytotoxic activity through increased levels of intracellular ROS arises from the generation of a highly reactive aquated form of cisplatin, which interacts with and depletes endogenous nucleophilic antioxidants such as reduced glutathione, methionine, and metallothioneins [3].

Many chemotherapeutic drugs, including cisplatin and erlotinib, are known to generate ROS and thereby increase levels of lipid derived aldehydes [4]. Lipid peroxidation refers to the oxidative degradation of lipid membranes, which generates reactive aldehydes, including 4-hydroxy 2-nonenal, malondialdehyde and acrolein, many of which are highly cytotoxic [5, 6]. Aldehyde dehydrogenase (ALDH) is a superfamily of 19 human isoforms that metabolizes reactive aldehydes produced endogenously and exogenously [4, 5, 7–9]. Thus, ALDHs play a critical role in metabolizing these reactive aldehydes and reducing oxidative stress in cells [10].

ALDH2 is a major enzyme responsible for detoxifying ROS produced from acetaldehydes derived from ethanol metabolism in the liver [11]. But, recently, the role of ALDH2 has been extensively investigated and expanded to include the detoxification of reactive aldehydes from drugs, food, spices, and from endogenous metabolism in tissues other than the liver, including heart, kidney, upper respiratory tissues, and brain [8]. ALDH2 variant is also important in the pathogenesis of multiples disease, including Fanconi anemia, osteoporosis, pain, diabetic complications, Parkinsons’s disease, Alzheimer’s disease, stroke, hypertension, myocardial infarction, cancer, and drug toxicity due to their vulnerability to ROS-induced damage [11].

Decreased ALDH2 activity due to ALDH2 mutation and the production of reactive oxygen species in the treatment of chemotherapy, however, have not been studied in detail. We have previously shown that small molecule ALDH inhibitors (“Aldis” for *aldehyde dehydrogenase inhibitors*) can increase the sensitivity of the lung cancer cell line A549 to the cytotoxic effects of mafosfamide (a metabolite of cyclophosphamide), possibly by inhibiting the metabolism of the chemotherapeutic drug into its inactive metabolite [9]. Similarly, reduced ALDH2 activity may not effectively detoxify ROS produced by cisplatin and may make tissues more susceptible to cisplatin-induced cytotoxicity.

Here, we sought to determine if ALDH2 mutation confers different chemosensitivity to cisplatin treatment, using ALDH2*1/*2 knock-in mice. ALDH2*1/*2 is a point mutation of E487K that leads to inactive and deficient ALDH2 enzyme activity in humans. Also, this mutation causes the Asian Alcohol Flushing Syndrome, and the mutation is prevalent in East Asian population in Korea, China, Japan, Singapore and Taiwan. We created the ALDH2*1/*2 knock-in mouse using the same mutation that mimics the human ALDH2 mutation [12]. The ALDH2*1/*2 knock-in mouse has the same phenotype as human ALDH2*1/*2 with elevated acetaldehyde levels after alcohol challenge [12]. These mice represent human ALDH2*1/*2 point mutation better than the ALDH2 knockout mice [8, 12].

Our data demonstrate that mice with ALDH2*1/*2 mutation show increased ROS production in certain tissues in response to cisplatin treatment and suggest that the amount of cisplatin used in the patients may need to be adjusted based on their ALDH2 variant profile so as to reduce serious side effects from cisplatin. This may be a clinically important factor considering that there are about ~540 million (~8%) of the world population with ALDH2*1/*2 variant, especially in East Asian and their descendants, with <50% of the wild types’ enzymatic activity [11].

## Methods

### ALDH2*1/*2 knock-in mouse

All animal experimentation protocols were approved by the Stanford University Animal Care and Use Committee. An ALDH2*1/*2 knock-in mouse model was developed in our lab by replacing the mouse wild type ALDH2 allele with a mouse E487K mutant ALDH2 allele by homologous recombination. The ALDH2*1/*2 knock-in mouse differs only by one single amino acid within the ALDH2 gene compared with the wild type mouse as in our previous publication [12]. All mutant animals used in this study are heterozygous ALDH2*1/*2 mice (referred to as ALDH2*2 from here on).

### Reagents

ROS assay was carried out using 2′,7′-dichlorodihydrofluorescein diacetate purchased from Sigma and Cell BioLabs (D6883, St. Louis, MO, and STA-342, San Diego, CA). Cisplatin was purchased from Enzo Life Sciences (ALX-400-040-M250, Ann Arbor, MI). MTT (3-(4,5-dimethylthiazol-2-yl)-2,5-diphenyltetrazolium bromide) reagent was purchased from Millipore (CT01-5, Temecula, CA).

### Reactive Oxygen Species (ROS) assay

Tissues were homogenized in lysis buffer (250 mM sucrose, 20 mM HEPES-NaOH, pH 7.5, 10 mM KCl, 1.5 mM MgCl2, 1 mM EDTA, 1 mM EGTA and protease cocktail inhibitor) and disrupted by hand-held homogenizer. The homogenates were spun at 800xg for 10 min and supernatant was collected as whole cell lysates or were spun again at 10,000xg for 15 min at 4 °C. The final cytosolic and lysates (containing mitochondrial fraction) were resuspended in 1% triton X-100. After the Bradford protein assay, cytosolic and lysates (containing mitochondrial fraction, 100 μl each with equal amount of protein) were incubated with 2′,7′-dichlorodihydrofluorescein diacetate (DCFH-DA), (20 μM-1 mM) at 37 °C in the dark for 30 min to 1 h. Then, cytosolic and lysates were incubated with PBS or cisplatin, according to their in vivo treatment, and lysed using the lysis buffer from the kit. 2′,7′-dichlorodihydrofluorescein (DCF) fluorescence was measured within 30 min using a BioTek FL-600 plate reader (BioTek Instruments, Winooski, Vt., USA) at 485 nm excitation and 530 nm emission wavelengths. Data were expressed in nM of DCF as calculated from the standard curves.

### In vivo assay

Three month old male ALDH2*2 knock-in mutant mice or wild type C57BL/6 mice were from a breeding colony from our lab at Stanford University. All mice were kept under standard temperature, humidity, and timed lighting conditions and were provided with mouse chow and water ad libitum. All animal experimentation protocols were approved by the Stanford University Animal Care and Use Committee. In both wild type and ALDH2*2 mutant mice, cisplatin was injected intraperitoneally, given once at 2 mg/kg/day dissolved in saline. Tissues were harvested two days after the injection, fractionated and were used for ROS and MTT assay.

### ALDH2 activity assay

Cofactor and substrate (NAD^+^ and acetaldehyde) were added in the reaction buffer and the increase in the level of NADH was observed over time by spectrophotometer. For a 200 μl assay, 90 μl of 100 mM NaPPi at final concentration of 50 mM NaPPi in water (pH 9.0 (M.W. 446)), 45 μl of 10 mM NAD^+^ (2.5 mM NAD^+^), 2.7 μl of 18 mM acetaldehyde (f.c., 250 μM), 20 μl of ALDH enzyme (100 μg protein) and 45 μl H_2_O were added and mixed. Absorbance (O.D.) was measured at A340 nm for 1-3 min (6.22 O.D. = 1 mmole of NADH measured with 1 cm width cuvette or in a 96 well plate). Readout is at mol NADH /min/mg protein. We used samples with no acetaldehyde as a blank control. The tissue homogenization buffer consisted of 1 ml of 1 M Tris HCl pH 8.0 (final concentration of 0.1 M Tris HCl), 0.1 ml of 1 M DTT (10 mM DTT (M.W. 154)), 2.3 ml of 87% glycerol (20% glycerol) and 6.5 ml of H_2_O with 0.1 ml of Trion X-100 (1%) with protease inhibitor.

### Colorimetric MTT assay for cell viability

MTT assay reagents from Millipore were used for cell viability. The assay was carried out according to the manufacturer’s instructions. Tissues were homogenized and 100 μl of the cells with equal amount of protein (100 μg each) were added per well in a 96-well plate. Cells were treated with 0.01 ml of MTT (Millipore CT01-5, 50 mg/ml in PBS) solution, and were incubated for 4 h at 37 °C in the dark for the cleavage of MTT to occur. Color development solution (isopropanol with 0.04 N HCl, 0.1 ml each) was then added and mixed thoroughly. Within an hour, absorbance was measured at 570 nm. Data are calculated as absorbances measured at 570 nm and were reported in arbitrary units and expressed as percent of control.

### Statistics

Data are expressed as mean ± SEM. Statistical analysis of *t*-test was used to compare the different number of samples analyzed by ROS, ALDH2 activity assay and MTT assay of cells from ALDH2*2 knock-in mice or wild type mice. A value *p* < 0.05 is considered to be significant.

## Results

### Higher level of ROS in the ALDH2*2 knock-in mice compared to wild type mice

**Fig. 1:**
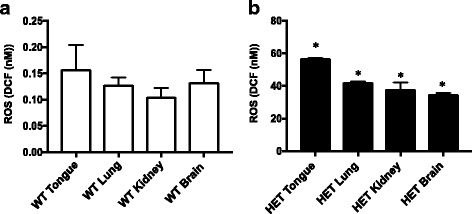
Higher level of ROS in the ALDH2*2 knock-in mice compared to the wild type mice. **a** Four different tissues (tongue, lung, kidney and brain) were isolated from C57BL/6 wild type (WT) mice, homogenized and the whole cell lysates were used to measure ROS levels using a reactive oxygen species (ROS) detection kit (Cell BioLabs, STA-342). The fluorescence intensity of 2′,7′-dichlorodihydrofluorescein (DCF) was measured within 30 min using a BioTek FL-600 plate reader (BioTek Instruments, Winooski, Vt., USA) at 485 nm excitation and 530 nm emission wavelengths. Data were expressed in nM of DCF as calculated from the standard curves using the standards of the kit. Results represent the means and SEMs of experiments with 3 samples each. **b** ROS level was measured from whole cell lysates of four different tissues of ALDH2*2 knock-in mice (referred to as HET from here on). Results are expressed as mean ± SEM (*p < 0.05 *vs*. respective wild type control, *t*-test, *n* = 6 each)

First, we used 3 month old, wild type mice that were untreated with cisplatin to measure the baseline level of ROS in these tissues. ROS levels were measured in the whole cell lysates of tongue, lung, kidney, and brain tissues (Fig. 1a). We observed levels of ROS in nM of 2′,7′-dichlorodihydrofluorescein (DCF) in tongue (0.16 nM), lung (0.13 nM), kidney (0.1 nM) and brain (0.13 nM). We focused on tongue, lung, kidney, and brain because cispatin is frequently used as a treatment for head and neck and upper respiratory tract cancers [1, 3] and also because ototoxiciy and nephrotoxicity are serious side effects of cisplatin [1, 3].

Then, we used 3 month old ALDH2*2 knock-in heterozygote mice with C57BL/6 background, which our group has previously developed [12]. When the ROS levels in ALDH2*2 knock-in mice were measured in the whole cell lysates from tongue, lung, kidney, and brain tissues, we observed a 200-400 fold increase in the ROS levels compared to those in wild type mice (Fig. 1b). There was a 350-fold increase in tongue (56 nM), 320-fold increase in the lung (40 nM), 380-fold increase in kidney (38 nM) and 260-fold increase in brain (34 nM). The absolute level of ROS was the highest in the tongue in ALDH2*2 knock-in heterozygote mice, which was significantly higher than all the other tissues screened.

### The wild type mice show no differences in the level of ROS when treated with cisplatin

**Fig. 2:**
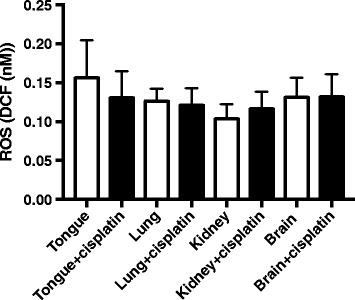
No differences in ROS levels in the wild type mice when treated with cisplatin. Wild type mice were treated with one cisplatin injection (2 mg/kg) and in two days, were euthanized and tissues were collected. Then, the whole cell lysates were analyzed for ROS levels. Results represent mean ± SEMs (*n* = 3 each)

When C57BL/6 wild type mice were treated with cisplatin, we did not observe increases in the ROS level in the whole cell lysates compared to PBS-treated ones (Fig. 2). Wild type mice were treated with PBS or cisplatin in vivo, and cells were isolated from the tissues. In the assay of ROS, the isolated cells were again treated with PBS or cisplatin, respectively, in vitro to amplify the effect of cisplatin treatment. While it is possible that ALDH2 protein expression may be different among the tissues, it seemed that the wild type ALDH2 activity was sufficient to detoxify the ROS load from cisplatin treatment.

### ALDH2*2 knock-in mice show lower level of ALDH2 activity in the kidney compared to wild type mice

**Fig. 3:**
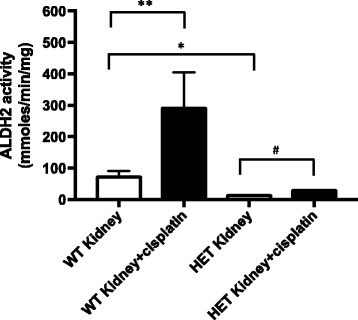
ALDH2*2 knock-in mice show lower level of ALDH2 activity in the kidney compared to wild type mice. Wild type and ALDH2*2 knock-in mice were treated with cisplatin, euthanized in two days and kidney tissues were collected. Cell homogenates were spun to isolate mitochondria (Figure 3) and ALDH2 activity was measured. Results represent mean ± SEMs (*p < 0.05 and **p < 0.05 *vs*. wild type (WT) control (*t*-test), and *# p <* 0.05 *vs*. PBS HET control (*t*-test))

To measure the ALDH2 activity in ALDH2*2 knock-in mice, we treated wild type and ALDH2*2 knock-in mice with cisplatin and compared ALDH2 activity in the kidney. We observed ~80% reduction in ALDH2 activity in ALDH2*2 knock-in mice compared to the wild type mice (Fig. 3). Interestingly, in the wild type mice, the level of ALDH2 activity increased by four fold in response to cisplatin compared to the untreated wild type mice, suggesting an induction of ALDH2 activity as a possible protective mechanism against ROS. Similar induction of ALDH3A1 was seen in cisplatin treated head and neck cancer cells (accepted manuscript, Kim et al., 2017). This induction of ALDH2 was also observed in cisplatin treated-ALDH2*2 knock-in mice compared to control mutant mice, but to a lesser degree (1.5 fold increase in cisplatin-treated mutant mice *vs*. 4 fold increase in cisplatin-treated wild type, compared to respective controls). This lower induction of ALDH2 activity in the mutant mice in response to cisplatin needs to be investigated further as this could exacerbate the nephrotoxicity in the patients with ALDH2*2 variant receiving cisplatin.

### ALDH2*2 knock-in mice show higher levels of ROS with cisplatin treatment

**Fig. 4:**
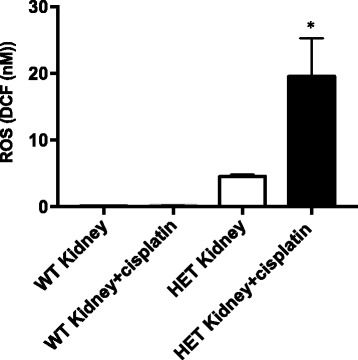
ALDH2*2 knock-in mice show higher levels of ROS with cisplatin treatment in the kidney cell lysates. ALDH2*2 knock-in mice were treated with cisplatin, euthanized in two days and tissues were collected. Cell homogenates were spun to isolate lysates, which include mitochondrial fraction (Figure 4) and ROS level was measured. Results represent mean ± SEMs (*p < 0.05 vs. PBS HET control (*t*-test))

To investigate the role of ALDH2 in conferring protection against ROS induced by cisplatin, we treated ALDH2*2 knock-in mice with cisplatin and compared the ROS levels in the tongue, lung, kidney and brain tissues. We first measured ROS in the cell lysates containing the mitochondrial fraction. We observed that only in the kidney, there was increased level of ROS observed with cisplatin treatment but not in other tissues (Fig. 4). Because it was reported that the ALDH2 gene is most highly expressed in the mouse liver but is in lower levels in the kidney, it is possible that the nephrotoxicity observed in humans is due to increased ROS from cisplatin treatment [1, 13] and that this toxicity could be exacerbated in patients with ALDH2*2 variant receiving cisplatin.

**Fig. 5:**
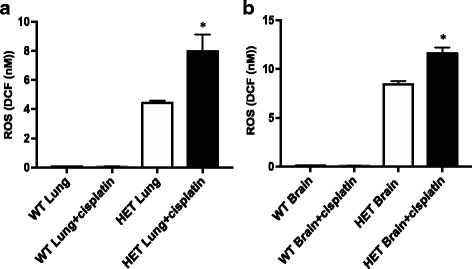
ALDH2*2 knock-in mice show higher levels of ROS with cisplatin treatment in the cytosolic fraction of lung and brain. ALDH2*2 knock-in mice were treated with cisplatin, euthanized in two days and tissues were collected. Cell homogenates were spun to isolate supernatant, which includes cytosolic fraction from lungs and brains (**a** and **b**) and ROS level was measured. Results represent mean ± SEMs (*p < 0.05 *vs*. PBS HET control (*t*-test))

When we measured ROS level in the cytosolic fraction of cells from tongue, lung, kidney, and brain, we observed a higher level of ROS in the lung and brain tissues (Fig. 5a and b) but not in other tissues. This increase in ROS with cisplatin treatment was only observed in ALDH2*2 mutant mice, but not in wild type mice (Fig. 2 *vs*. 4 and 5). It is possible that there is activation of different ALDH isozymes in the cytosolic fraction of these tissues than in the mitochondrial fraction to detoxify ROS load, such as cytosolic forms of ALDH, e.g., ALDH1A1, 1A2, 1A3, 3B1, 8A1 or 9A1 [11]. It is also possible that other phase 1 oxidizing enzymes, which ALDH is also part of, may have increased in the cytosolic fraction but this warrants further investigation in the future. These data suggest that the defect in ALDH2 activity in the ALDH2*2 knock-in mutant mice plays a part in increasing the ROS level with cisplatin treatment and that the effect could be tissue-specific, which needs to be explored further.

### ALDH2*2 knock-in mice show higher cytotoxicity in the kidney with cisplatin treatment compared to wild type mice

**Fig. 6:**
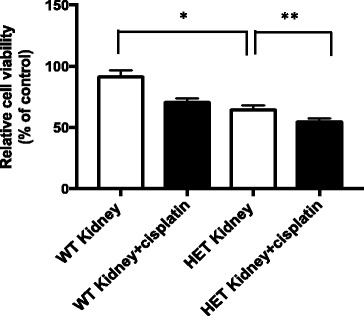
ALDH2*2 knock-in mice show higher cytotoxicity to cisplatin treatment in the kidney compared to wild type mice. Wild type and ALDH2*2 knock-in mice were treated with cisplatin, euthanized in two days and kidney tissues were collected. Cells from whole cell homogenates (100 μg protein, each) were used and cell proliferation was measured using MTT assay (Figure 6). Results represent mean ± SEMs (*p < 0.05 *vs*. wild type (WT) control and **p < 0.05 *vs*. control ALDH2*2 knock-in mice (*t*-test), *n* = 7-8 each)

To investigate the cytotoxicity to cisplatin treatment in wild type and ALDH2*2 knock-in mice, we treated mice with cisplatin and compared viability of the kidney cells from lysates using MTT assay. We observed 30% reduction in the kidney cell viability in ALDH2*2 knock-in mice compared to the wild type (Fig. 6). With cisplatin treatment, there was further decrease in cell viability by 10% compared to the ALDH2*2 knock-in control (Fig. 6). The reduction in cell viability in the cisplatin treated ALDH2*2 knock-in mice compared to the control ALDH2*2 knock-in mice, suggest that ALDH2 deficiency could increase cisplatin-induced cytotoxicity due to the inefficient removal of ROS.

## Discussion

ALDH isozymes are responsible for detoxifying intracellular reactive aldehydes and protecting cells from ROS-induced oxidative insult. However, the role of ALDH2, in particular, in cisplatin chemosensitivity has not been investigated in detail. We hypothesized that lower ALDH2 activity can effectively increase the oxidative insult from cisplatin.

Other types of ALDH subtypes, for example, ALDH1, alone or with the expression of cell surface stem cell markers CD44 or CD133, has been used to enrich a cell population with chemoresistant and stem-cell like properties in head and neck squamous cancer cells [14]. Previously, ALDH2*2 mutation was shown to increase susceptibility to upper respiratory tract and head and neck cancers [15]. Here, we demonstrate that ALDH2*2 mutation with reduced ALDH2 activity resulted in a dramatic increase in ROS level in the tongue, lung, kidney, and brain as compared with wild type mice. In addition, cisplatin treatment in ALDH2*2 mutant mice further elevated the level of ROS, suggesting a functional role for this isozyme in cisplatin sensitivity. The fact that ALDH2*2 mutation is a double edged sword that increases both susceptibility to certain upper respiratory tract/esophageal cancers and can also limit the amount of cisplatin, may be useful information in the context of cisplatin treatment.

We sub-fractionated tissues into cell lysates containing mitochondrial and cytosolic fractions, respectively, to evaluate the difference in ROS level in different cellular fractions, though ALDH2 is known to be located in the mitochondrial matrix [11]. It is possible that the increases in the level of ROS in the lysates of kidney as well as in cytosol of lungs and brains are due to lower amount of ALDH2 protein and gene expression in these tissues compared to other tissues, like in the liver [16, 17]. In humans, ALDH2 gene is highly expressed in high metabolic organs, such as liver, muscle, heart and in kidney [16, 17]. Quantitative analyses of the protein level or the activity of different ALDH isozymes in response to cisplatin is warranted to further understand the differences in ROS level in different tissues and cell fractions of the ALDH2*2 knock-in mouse.

When we measured cell viability in the kidney from wild type and ALDH2*2 knock-in mouse without additional in vitro cisplatin treatment (Fig. 6), we observed increased cytotoxicity in the ALDH2*2 knock-in mouse compared to the wild type, possibly due to the increases in the ROS level. Further increases in cytotoxicity was observed in cisplatin-treated ALDH2*2 knock-in mouse compared to control mutant mouse (Fig. 6). This increased cytotoxicity to cisplatin in mutant mice could be a clinically important concern in ALDH2 variant population with less than 50% of the wild type’s enzyme activity.

In this study, we investigated the level of ROS in the wild type mice and compared those from the ALDH2*2 mutant knock-in mice. Our data indicate that specific tissues may be impacted by cisplatin in different ways depending on their ALDH2 expression level and activity. It is possible that other ALDH isozymes, different oxidizing enzymes or antioxidants available can affect the outcome of cisplatin treatment. An increase of cisplatin-induced DNA damage was reported in head and neck squamous cell carcinoma (HNSCC) cells with reduced ALDH2 activity [18]. To our knowledge, there is not much further data available on the role of ALDH2 and susceptibility to cisplatin-induced ROS damage to cells. In the future, the viability of ALDH2-deficient cancer cells, or in a cancer model with both male and female mutant mice treated with cisplatin, would be an appropriate extension of this work and will provide important data to understand the functional role of ALDH2, especially in the context of cancer treatment.

## Conclusion

Here, we observed that, when treated with cisplatin, ALDH2*2 knock-in mutant mice showed increased production of ROS in the kidney, lung, and brain compared to wild type mice with normal ALDH2 activity. We also observed that, when treated with cisplatin, ALDH2*2 knock-in mutant mice showed increased cytotoxicity in the kidney compared to mutant control. This suggests that ALDH2 activity, in a tissue-specific manner, may confer different levels of susceptibility to ROS-induced cisplatin cytotoxicity and may need to be considered in the treatment of cisplatin in patients.

## Abbreviations

ALDH: Aldehyde dehydrogenase
ROS: Reactive Oxygen Species
